# Prioritisation of head, neck, and respiratory outcomes in mucopolysaccharidosis type II: lessons from a rare disease consensus exercise and comparison of parental and clinical priorities

**DOI:** 10.1186/s13023-025-03581-y

**Published:** 2025-02-26

**Authors:** James Dempsey, Jessica Daniels, Roulla Katiri, Sophie Thomas, Aleksandra Metryka, Mira de Kruijf, Stuart Wilkinson, Simon A. Jones, Iain A. Bruce

**Affiliations:** 1 Paediatric ENT Research, Royal Manchester Children’s Hospital, Manchester University NHS Foundation Trust, Manchester, M13 9WL UK; 2 Division of Infection, Immunity and Respiratory Medicine, School of Biological Sciences, Faculty of Biology, Medicine and Health, University of Manchester, Manchester, UK; 3 Manchester University NHS Foundation Trust, Manchester, UK; 4 Hearing Sciences, Mental Health and Clinical Neurosciences, School of Medicine, University of Nottingham, Nottingham, UK; 5 Royal National ENT & Eastman Dental Hospitals, University College London Hospitals NHS Foundation Trust, London, UK; 6 MPS Society, MPS House, Repton Place, Amersham, Buckinghamshire UK; 7 Research & Innovation, East Lancashire Hospitals NHS Trust, Royal Blackburn Teaching Hospital, Blackburn, UK; 8 Department of Paediatric Otorhinolaryngology, Royal Manchester Children’s Hospital, Manchester Academic Health Science Centre, Manchester, UK

**Keywords:** Mucopolysaccharidosis type II (MPS II), Core outcome set, Head, neck, and respiratory disease, Outcome measures, Patient and public involvement, Rare diseases

## Abstract

**Background:**

The mucopolysaccharidoses are a group of rare, inherited metabolic disorders. MPS II is a X-linked recessive disease, also known as Hunter syndrome. Clinical manifestations include upper and lower respiratory tract, and head and neck pathologies influencing quality of life, morbidity, and mortality. Medical and surgical intervention outcomes for MPS are reported inconsistently, creating a challenge when synthesising and contrasting evidence. This study set out to address the inconsistency in outcome measurement in this field. International recommendations for developing a core outcome set were adopted. Available data from qualitative studies and outcomes from a modified e-Delphi surveys were used to develop a list of candidate outcomes for consideration. Three consensus meetings with patients diagnosed with MPS II alongside their parents/carers were ran to help finalise a list of outcome domains.

**Results:**

Survival, airway obstruction, and quality of life were outcomes identified as important to always measure in all MPS II clinical trials and/or in clinical practice. Other outcomes for younger children included swallowing difficulties, cognitive development, ability to participate in education, and communication. The adolescent group included safety of chewing and swallowing, complications of anaesthesia, sleep quality and apnoea, nasal problems, and chronic otitis media. The adult group identified sleep apnoea, and hearing, as additional outcomes to measure.

**Conclusions:**

A novel methodology for determining a core outcome set in rare diseases has been recommended. Both functional and quality of life outcomes were identified by the three age groups of individuals and/or their parents. Adoption of these sets of outcomes in future clinical trials and/or clinical practice will enable comparison of outcomes reported.

## Background

The mucopolysaccharidoses (MPS) are a group of rare, inherited metabolic disorders caused by deficiencies in specific lysosomal enzymes responsible for glycosaminoglycan (GAG) degradation [1]. Glycosaminoglycans are ubiquitous macromolecules, present on the cell surface, inside the cell, and within the extracellular matrix (ECM). The function of GAGs, once thought to be solely concerned with cell hydration and scaffolding, is now understood to be much more complex [1]. Playing an integral role in cell–cell interaction, cell adhesion and signaling, as well as a host of other biological activities [2]. Eleven enzyme deficiencies are responsible for seven different diseases phenotypes (MPS I, II, III, IV, VI, VII and IX) with clinical and molecular characterization of a novel subtype (subtype X) being described recently [3, 4].

MPS II is an X-linked recessive disease, also known as Hunter syndrome. It is characterised by a deficiency of the enzyme iduronate-2-sulphatase. This enzyme is responsible for the first step in the metabolism of dermatan and heparan sulphate, both found throughout the body [5]. As such, the clinical manifestations are both multi-systemic and progressive, with involvement of the upper and lower respiratory tracts contributing significantly to morbidity and mortality [6, 7]. MPS II is often divided into neuronopathic and non-neuronopathic although there is no clear binary distinction between these forms and it is now better understood as a full spectrum [8, 9].

Although initial MPS symptoms can be subtle and non-specific, the involvement of head and neck structures is an early and near-universal characteristic. This includes adenotonsillar hypertrophy, upper airway obstruction, macroglossia, airway GAG deposits, and tracheomalacia [10]. Lower respiratory function is compromised by thickened secretions, luminal obstruction, and restrictive pathology due to musculoskeletal anomalies of the neck and chest [11]. Hearing loss is a common symptom of MPS II and a consequence of an increased development and persistence of otitis media with effusion [12, 13].

MPS II has an estimated incidence rate ranging from 1 in 100,000 to 170,000 live births, and is the only X-linked MPS [14]. The introduction of newborn screening has suggested that the prevalence of MPS II may be higher than previously thought. However, newborn screening for MPS has only been implemented in a small number of geographical locations [14, 15]. Due to its rarity, there is a paucity of good-quality data to support evidence-based management [16, 17]. Currently, the standard approved treatment is enzyme replacement therapy (ERT), with gene therapy and fusion-ERT in experimental stages [18]. ERT aims to improve the course of the disease by reducing somatic symptoms. Data available originates from tertiary centre case series, with heterogeneity in outcome selection and reporting [11].

Selecting appropriate outcomes is of paramount importance in the design of clinical trials, ensuring that outcomes considered important by stakeholder groups are reported on, and to avoid research waste [19]. Homogeneity in outcome selection facilitates the combination and contrasting of data to increase the strength of available evidence. The COMET (Core Outcome Measures in Effectiveness Trials, https://www.comet-initiative.org/) initiative has developed recommendations aiming to standardise reporting in clinical trials to facilitate the synthesis of high-quality evidence to guide clinical practice [20]. Additionally, standardising reported outcomes reduces duplication of study methodology and outcome reporting bias [21].

Following COMET recommendations, a core outcome set (COS) is being developed for the MPS collectively [22]. This study represents the first attempt to develop an organ system-specific COS, focused on the leading cause of morbidity and death in an MPS subtype. The developed COS could subsequently be modified for use in other subtypes of MPS. A recent qualitative systematic review highlighted the need for a more holistic, coordinated care approach [23]. In preparation to develop a COS specific to MPS II, a tailored qualitative study [24], and a systematic review [25], were undertaken to identify a ‘long list’ of candidate outcomes. This long list was ratified using a modified Delphi methodology to develop a consensus on a COS for head, neck, and respiratory disease in MPS II.

## Methods

**Fig. 1:**
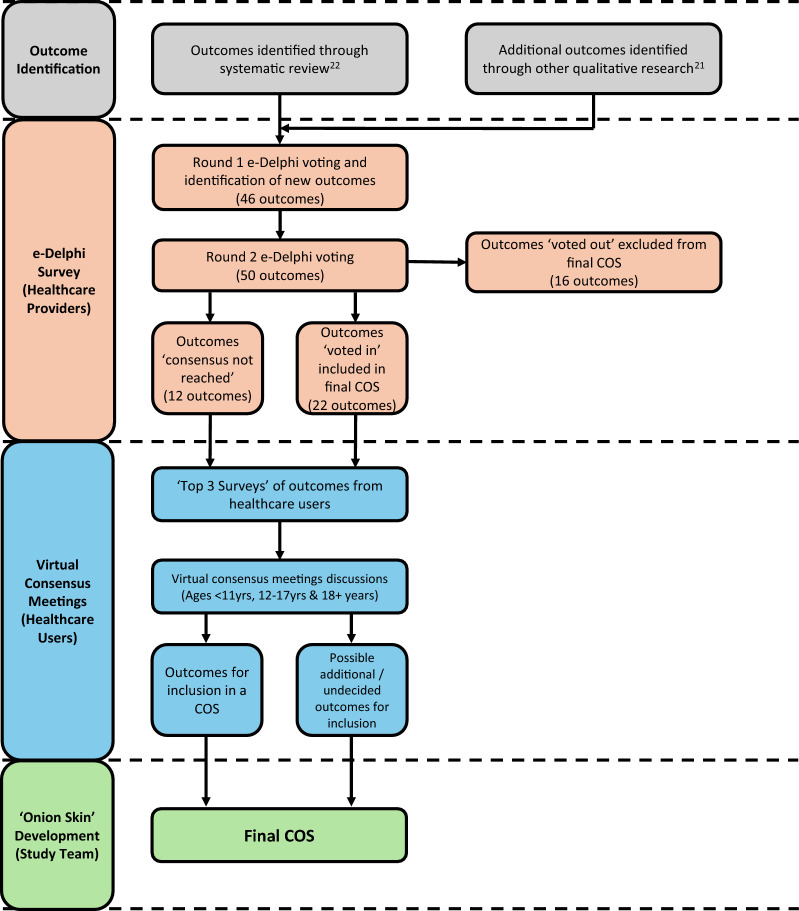
Overview of the methodology adopted by the COHERE study group

The Core Outcome set for Head, nEck and REspiratory disease in MPS II (COHERE) study follows the methodological principles set out from the recommendations developed by the COMET initiative [20]. Methods included a modified electronic Delphi (e-Delphi) process to achieve consensus of opinion among healthcare users and professionals working in the field internationally. Several challenges were faced during the development of this COS, such as limited healthcare providers with sufficient experience in the field, and limited engagement from healthcare users. As such, we had to modify the COS methodology in real-time. Therefore, a novel approach to developing a COS in a rare disease has been implemented. An overview of the methodological process adapted by the COHERE group can be seen in Fig. 1. The COHERE group therefore suggest a modified methodological process when determining a COS for a rare disease.

### Outcome identification

To develop the ‘long list’ of potentially important candidate outcomes, a preceding systematic review of the literature and clinical trials registry search was conducted [25]. Additionally, themes identified by qualitative research performed by Soni-Jaiswal were added [24]. From these sources, 46 outcomes were identified and grouped according to an existing outcome taxonomy [26]. Each of the 46 candidate outcomes was accompanied by a plain language definition (Appendix 1). Outcomes and definitions were reviewed and approved by the members of the study management group (SMG) and were presented to all the stakeholder representatives.

### Stakeholder participants

The COHERE study aimed to consider the views of patients, families and/or carers, healthcare professionals, and clinical researchers working in the field. Healthcare professionals involved in the care of patients with MPS II were invited to participate, which included otolaryngologists, respiratory physicians, clinical geneticists, metabolic physicians, specialist nurses, physiotherapists, general paediatricians, and audiologists. Inclusion and exclusion criteria are below.

### Inclusion criteria

Healthcare users:

- Confirmed MPS II diagnosis
- Aged between 12 and 25 years old

Parents and/or carers:

- Parents and/or main caregivers of children with MPS II
- Adults aged 18 or over

Healthcare professionals:

- Clinicians responsible for the direct care and management of at least two patients with MPS II in the last 12 months

### Exclusion criteria

Healthcare users:

- Unconfirmed MPS II diagnosis
- Aged below 12 and above 25 years old

Parents and/or cares:

- Not directly involved in day-to-day care

Healthcare professionals:

- Clinicians who only occasionally or never looked after patients with MPS II

### Recruitment

Recruitment was facilitated by professional networks and the MPS Society [27], reaching out to international MPS societies to advertise the study. Although there is no required sample size for an e-Delphi survey [28, 29]; the aim was to recruit at least 40 participants in total for the e-Delphi survey. One of the key-deciding factors for participant recruitment is that the participant panel membership should adequately represent corresponding stakeholder groups [20].

Purposive sampling methods were used to recruit healthcare professionals who were experts in metabolic disorders. Clinicians received group electronic advertisements via professional groups and/or personal contact from the study advisory group (SAG). Healthcare users received a group electronic advertisement with the study information through a trusted patient group (MPS Society, MetabERN [30]). Those who decided to take part were directed to the e-Delphi webpage which included detailed study participant information. If the participant agreed to take part, a link was provided for the online DelphiManager, a software maintained by the COMET initiative (https://www.comet-initiative.org/delphimanager/). The participants agreed to a series of consent statements prior to being able to proceed to the survey. Demographic information was also recorded, dependent upon the stakeholder group of the participant (Appendix 2).

### e-Delphi survey

Delphi surveys have been used extensively for the development of COS [31]. To prioritise the outcomes to be included in the COS internationally, healthcare users and professionals were invited to take part in two modified e-Delphi surveys. Participants were not offered payment or vouchers for their time to take part in the e-Delphi.

### e-Delphi survey—Round 1

Participants were presented with a choice of two e-Delphi surveys: for the age group 0–11 years old and another for 12–25 years old. Participants were allowed to complete both surveys if they considered themselves to have appropriate experience. Participants were asked to score each outcome domain on a 9-point scale proposed by the Grading of Recommendations Assessment, Development and Evaluation (GRADE) group [32]. Scores of 1 to 3 signified an outcome of ‘limited importance’, a score of 4 to 6 signified ‘important but not critical’ outcome, and a score of 7 to 9 signified a ‘critical’ outcome. Participants were also given an ‘*unable to score’* option and could comment on any aspects of the scoring or outcome domains using open-text boxes. Round 1 participants were also able to suggest additional outcomes that they considered important. Suggested outcomes deemed to represent a new outcome domain by the SMG and discussed with the SAG were added to the list for consideration in round 2. Round 1 was open for approximately 6 months, with analysis of the responses lasting approximately one month prior to opening round 2 for voting.

### e-Delphi survey—Round 2

Due to low level of engagement from parents/carers and patients, our methodology had to be modified. Therefore, round 2 was opened to healthcare providers only. Attempts were made to address the lack of participation from healthcare users by employing comprehensive support from an established information and news resource, the MPS Society. Both the MPS Society and the investigative team believed it was crucially important to include the perspectives of those with ‘lived experience’ in the final COS, as per established recommendations [33]. Therefore, perspectives of healthcare users would be gathered during the consensus meeting stage of the study.

All items, including additional outcome domains suggested by participants in round 1, were carried forward for consideration in round 2. Descriptive statistics were used to summarise the scores from round 1 and presented to round 2 participants. Participants could see the results of their individual score for each outcome in addition to the average score of each stakeholder group. The rationale for this approach was that it may improve consensus between the stakeholder groups [34]. Round 2 was opened for approximately 4 months, with response analysis lasting up to one month.

### Missing responses

If a participant did not complete a subsequent round of the Delphi survey, their scores from previous rounds were counted as valid and retained in the study. Similarly, if a participant failed to score a specific item during a survey round, the answers to other items were held as valid and retained as long as the participant scored over 50% of all of the outcomes. Participants that failed to score over 50% of outcomes in round 1 were excluded from analysis and not contacted for round 2. This criterion has been successfully utilised by other COS development teams and ensured that participants contributing to the decision-making were fully engaged in the process and had sufficient expertise [28, 35].

### Analysis of the e-Delphi surveys

Scores from round 2 were analysed using descriptive statistics and outcomes were grouped into the following categories, as per COMET initiative recommendations [21]:

1. Consensus reached for inclusion in COS: outcomes scored as ‘critically important’ by greater than 70% of participants *and* ‘unimportant’ by less than 15% of round 2 participants,
2. Consensus reached for exclusion from COS: outcomes scored as ‘critically important’ by less than 50% of round 2 participants, and
3. Consensus not reached: any other combination of scores.

Outcomes that were ‘voted out’ were not carried forward and not included in the final COS (Fig. 1). The rationale for these categories was that for an outcome domain to be included in the COS, it requires agreement by the majority regarding the critical importance of the outcome, with only a small minority considering it to have little importance.

### Consensus meetings

Integrating the opinions of experts by experience (i.e., healthcare users) is fundamental in developing a COS in a rare disease. Making COS development meaningful and accessible for healthcare users involves them having a genuine say in the development process [33]. The COHERE team were faced with lack of engagement from healthcare users during the e-Delphi stages. The challenge in securing meaningful input from healthcare users on an international scale was addressed by capturing the thoughts of healthcare users during the consensus meetings.

With the aid of the MPS Society, three virtual meetings were planned for differing age groups and hosted over Zoom [36]. All international MPS Societies and the Muenzer MPS Center [37] were contacted and asked to broadcast advertisement for the consensus meetings in an aid to boost international collaboration. The participant groups incorporated adults over 18 years of age (Group 1), teenagers aged 12–17 years old (Group 2), and children younger than 11 years old (Group 3). Parents or carers of the participants were invited to contribute for groups 2 and 3 also, and, if needed, in group 1.

In advance of the consensus meetings, participants revisited the outcomes and selected the three they deemed most important from the ‘voted in’ or ‘consensus not reached’ categories following the e-Delphi surveys (Fig. 1). This approach was successfully utilised in the development of a COS for single-sided deafness [28]. The identified outcomes were discussed during the corresponding consensus meeting to determine their inclusion or exclusion from the COS.

The consensus meetings provided opportunity for semi-structured discussions lasting approximately two hours. Discussions amongst participants were encouraged based on personal opinion and experience. Anonymised voting was conducted using the Zoom polling function to pre-determined questions. Results were presented to participants using histograms generated by Zoom. All consensus meeting discussions were recorded for analysis, recordings were deleted once this was completed.

### ‘Onion skin’ development

OMERACT (Outcome Measures in Rheumatology) principles were used when determining the classification of the final outcomes included in the agreed COS [38]. Hence, outcomes ‘voted in’ during the e-Delphi were included in the final COS. Likewise, outcomes identified for inclusion or those undecided by the consensus meetings were also included in the final COS. This methodology encompasses the perspectives of both healthcare professionals and healthcare users with expertise in MPS II by profession or experience. Following OMERACT principles, the selected outcomes were categorised as (1) mandatory outcomes; (2) mandatory outcomes for specific circumstances; (3) important but optional outcomes; and (4) research agenda outcomes. We acknowledge that the group of children and young people with MPS II is diverse and may have varying priorities depending on age, life stage, and health status, therefore a COS would be determined for each of the age groups separately.

## Results

### e-Delphi surveys

A total of 38 votes were obtained from healthcare professionals, and seven parental votes during the first round of the e-Delphi. Due to low healthcare user participation during round 1, healthcare users were withdrawn from the e-Delphi survey phase of the COS development because meaningful and robust results analysis was not possible. During round 1 four new outcomes were suggested by participants and were presented to voters for consideration during round 2. These were (1) ability to attend school/college or to seek employment, (2) ability to secure the airway by endotracheal intubation, (3) behaviour, and (4) sleep quality.

As such, 50 outcomes were presented to healthcare professionals for voting in round 2 of the e-Delphi. Analysis of round 1 voting led to the amalgamation of the two separate age groups of the e-Delphi (0–11 and 12–25), creating one of 0 to 25 years of age for round 2 of the e-Delphi. Registration data showed that some clinicians voted in both surveys. The Delphi Manager software (https://www.comet-initiative.org/delphimanager/) used anonymises participants, therefore, it was not known which votes belonged to the same participant.

Significant voter attrition was noted between rounds 1 and 2, with only 15 votes being made in round 2 compared to 38 in round 1 (39.47% of healthcare professional participants). As this was an online exercise it was not possible to gather reasons as to why participants did not take part in round 2. Consensus was reached for 22 outcomes to be included (Table 1), 16 outcomes to be excluded (Table 2) and consensus was not reached for 12 outcomes (Table 3).

**Table 1:** List of outcomes ‘voted in’ at e-Delphi stage, for inclusion in the final COS

Dodd taxonomy core area	Outcome	% voted ‘not important’	% voted ‘important’	% voted ‘critically important’
Mortality/survival	Survival	0	14.3	85.7
Physiological/clinical	Airway obstruction	0	0	100
	Hearing	0	7.7	92.3
	Sleep apnoea	0	7.7	92.3
	Dyspnoea	0	7.7	92.3
	Pneumonia	0	14.3	85.7
	Swallowing difficulties	0	15.4	84.6
	Enlarged tonsils	0	21.4	78.6
	Enlarged adenoids	0	21.4	78.6
	Bronchitis	0	23.1	76.9
	Chronic cough	0	28.6	71.4
Life impact	Quality of life	0	0	100
	Communication skills	0	14.3	85.7
	Emotional impact of the disease on the patient	0	21.4	78.6
	Emotional impact of the disease on the parent/carer	0	21.4	78.6
	Language development	0	28.6	71.4
Resource use	Prevalence of pulmonary-related hospitalisations	0	7.1	92.9
	Need for non-invasive ventilatory support	0	7.7	92.3
	Tracheotomy	0	7.7	92.3
	Need for oxygen therapy	0	7.7	92.3
Adverse events/effects	Complication of surgical treatment	0	15.4	84.6
	Ability to secure the airway by endotracheal intubation	0	23.1	76.9

**Table 2:** List of outcomes ‘voted out’ at e-Delphi stage, for exclusion from the final COS

Dodd taxonomy core area	Outcome	% voted ‘not important’	% voted ‘important’	% voted ‘critically important’
Physiological/clinical	Tympanic membrane perforation	0	53.8	46.2
	Purulent ear discharge	0	53.8	46.2
	Nasal polyps	0	63.6	36.4
	Blood gas	7.7	61.5	30.8
	Chronic nasal discharge	0	69.2	30.8
	Rhinorrhoea	0	69.2	30.8
	Rhinosinusitis	0	75	25
	Temporal bone status	8.3	75	16.7
	Tinnitus	0	92.3	7.7
	Vertigo	0	92.3	7.7
Life impact	Intelligence	0	57.1	42.9
	Education	0	64.3	35.7
Resource use	Non-surgical intervention to ears	0	53.8	46.2
	Need for surgical intervention to ears	0	61.5	38.5
Adverse events/effects	Behaviour	0	53.8	46.2
	Voice quality	0	69.2	30.8

**Table 3:** List of outcomes where ‘consensus was not reached’ at e-Delphi stage regarding inclusion or exclusion in the final COS

Dodd taxonomy core area	Outcome	% voted ‘not important’	% voted ‘important’	% voted ‘critically important’
Physiological/clinical	Chronic Otitis Media (COM)	0	30.8	69.2
	Pulmonary function	0	30.8	69.2
	Nasal congestion/obstruction	0	35.7	64.3
	Acute Otitis Media (AOM)	0	38.5	61.5
	Middle ear function	0	38.5	61.5
Life impact	Exercise tolerance	0	33.3	66.7
	Ability to attend school	0	38.5	61.5
	Independence	0	46.2	53.8
	Psycho-social development	0	50	50
Resource use	Tonsillectomy	0	38.5	61.5
	Adenoidectomy	0	38.5	61.5
Adverse events/effects	Sleep quality	0	30.8	69.2

### Consensus meetings

Five participants took part in each of the consensus meetings with attendees residing in either the United Kingdom (UK) or the United States of America (USA). An overview of the consensus meetings details, as well as outcomes discussed and included within the COS are listed in Table 4.

**Table 4:** Summary of consensus meetings and included outcomes in a core outcome set

	Group 1—Adults (18–25 years old)	Group 2—Teenagers (12–17 years old)	Group 3—Children (under 11 years old)
Meeting date	17th October 2023	14th March 2024	26th September 2023
Number of participants	3 Parents and/or carers 2 Healthcare users	5 Parents and/or carers	5 Parents and/or carers
Selected Outcomes for Inclusion in a COS	*Airway obstruction* (to incorporate tracheotomy)	Airway obstruction*	*Airway obstruction*
	*Quality of life* (to include the emotional impact of the disease)	*Quality of life*	*Quality of life*
	*Survival*	*Survival*	*Survival*
	Sleep Apnoea	Sleep Apnoea	Lower respiratory problems /Lung disease*
	Communication*	Sleep quality	Communication (receptive/expressive)*
	Hearing	Swallowing difficulties (to include safety and chewing)	Swallowing difficulties
		Nasal congestion/obstruction	Cognitive development
		Chronic Otitis Media	
		Complications of anaesthesia*	

Outcomes highlighted in italics show common outcomes between all three groups. Outcomes marked with an asterisk are outcomes which were identified as possible additional/undecided outcomes for inclusion

**Fig. 2:**
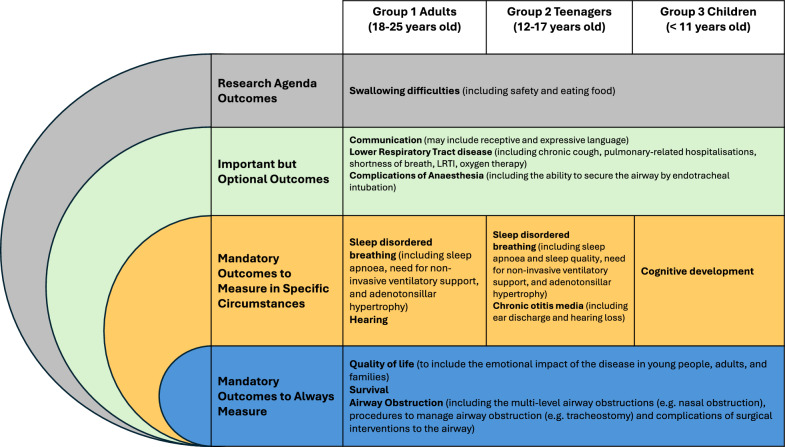
MPS II core outcome set. Adapted from the OMERACT onion skin [39]

Following the consensus meetings a final COS was determined for each of the three age groups generated from the three consensus meetings and followed the guidelines provided by the OMERACT and COMET groups [20, 39]. Outcomes pertaining to similar domains or pathologies have been grouped as determined by the SMG. The final COS for each age group can be seen in Fig. 2.

### Participant qualitative input

Survival was agreed ubiqitously by all three groups to be included in the final COS as an essential outcome to measure. The importance of this outcome is demonstarted by this quote by a parent of teenagers with MPS II:

“…every morning, and I’m sure we all could relate to this. When I wake up, I’m the first one to get up in the morning. I walk into the bedrooms and I poke them just to make sure that they’re still alive” (Group 2 participant).

Similary, airway obstruction was voted by all groups to be included in the COS as demonstrated by the following quote by an adult participant with MPS II:

*“…(child) had a tracheostomy at 11 years…is now 24 years, think it was the best thing that could of happened…think it’s probably prolonged his life, has a good quality of life, enables him to get out and about, I didn’t want it but was the best thing*” (Group 1 participant).

Following discussions at the consensus meetings, the airway obstruction definition was modified to encompass nasal obstruction, management of the airway obstruction (e.g., tracheotomy), and complications of surgical interventions to the airway.

Both Groups 1 and 3 consensus meetings included lengthy discussions around independence. Ultimately, it was decided by adult participants in Group 1 that independence does not need to be included in the final COS, as it did not seem necessary in relation to this specific COS. Conversely, a more in-depth conversation was held in Group 3. Following on from the results of the pre-consensus meeting ‘top 3’ prioritisation survey the outcomes independence and ability to attend school or participate in education were discussed. Suggestions were then made to encompass both of these outcomes within the term ‘cognitive development’:

*“…cognitive development would be easier to measure separately with some sub categories…with independence falling underneath this*” (Group 3 participant).

Varying opinions were expressed regarding communication as an outcome. In Group 1 some participants felt it was not relevant to clinical trials, whilst others believed communication is an invaluable outcome to measure:

*“…for me I would want to tell you how, if I’m in pain, emotionally stable. Past experience, if you had no communication you couldn’t be involved*” (Group 1 participant).

Group 3 discussed further subcategorisation of communication as receptive and expressive:

*“…communication is more appropriate term but children have different communication. Some are verbal, some are non-verbal. So many ways to communicate, make it more broad, separate out receptive and expressive*” (Group 3 participant).

## Discussion

This study completes the first step in the development of a commonly agreed COS for head, neck, and respiratory manifestations of MPS II. Despite the challenge of bringing international stakeholder representatives of a rare disease together, a set of commonly agreed outcomes were deduced for measurement in future clinical trials and/or in clinical practice. Adoption of this COS by healthcare professionals and clinical researchers will promote consistency of measurement and reporting of effect following therapeutic interventions for MPS II.

This work complements efforts by Howie and colleagues who are developing a COS for all subtypes of MPS in children aged under 18 years [22]. The COHERE study is complementary in aiming to identify head, neck, and respiratory outcomes in young children, teenagers, and young adults with MPS II. By using similar methodologies, we can strengthen the evidence for choice of outcomes to measure in an MPS sub-type. Comparison of the COS developed by Howie and colleagues will be interesting to check for consistency of stakeholder opinions.

The outcomes derived for this study were grouped following recommendations from Dodd and colleagues [26]. The advantage of using this taxonomy is that it is not disease-specific, it is comprehensive, it has been developed for trial outcomes, and has successfully been utilised in other studies developing COS [28, 40, 41]. Delphi surveys have been used to develop COS due to their anonymity and minimising response bias. Other benefits include the ability to give all participants an equal voice in scoring and prioritising outcomes [42].

Survival was an outcome domain that was unanimously included by all participant groups. Survival data is also recommended by the Cochrane handbook, suggesting that adverse events are rigorously monitored and reported on when synthesising evidence for interventions [43]. Despite these recommendations, our systematic review [25] demonstrated that survival is not always reported.

Airway obstruction (including tracheotomy), ability to intubate, and sleep apnoea were included in the final COS for all participating age groups. These outcomes are reported by clinical researchers, but reporting methods vary between studies creating inconsistency in reporting and making data synthesis in metanalyses challenging [25]. This demonstrates a lack of suitable outcome measurement instruments (OMI) and stresses the importance of OMI development to enable the consistent reporting of these mandatory outcomes as identified by this COS.

### Strengths and limitations

Although COMET methodology was used here, modifications were required due the challenges facing qualitative research into a rare disease. Namely, adequate recruitment of stakeholders and co-ordinating different stakeholder groups on different continents, across differing time zones. Despite combined efforts with the MPS society to engage healthcare users, it was not possible to recruit significant numbers of healthcare users during the Delphi phase. This may suggest that e-Delphi surveys are not the optimal methodology to engage patients, parents and/or carers. Patients and parents within the field of rare diseases are more familiar with focus groups. Therefore, virtual or face-to-face consensus meetings may be more acceptable for these healthcare users. The MPS society was invaluable in the recruitment of participants and as a trusted source of information and point of contact for both the study team and participants. While we did not differentiate between MPS II subjects based on central nervous system involvement or not, this is challenging to define and currently lacking in consensus especially when self-reported. The feature may or may not alter perceptions of key core outcomes.

Such consensus meetings can be facilitated in different languages, aided by translators, and can span various communities and/or countries to ultimately promote and expand participation. Despite efforts to fully represent stakeholders internationally (e.g., advertising on social media, using MPS Society contacts), all participants were from the UK or USA, and there was no participant recruitment from low- and middle-income countries. Hence this study did not require translators or translated documents to proceed. This is a commonly reported challenge in COS development, but it is acknowledged that geographical and socio-economic differences should be considered [44]. Other studies for more common healthcare interventions, like the GASTROS study, set up surveys in multiple languages and opened in numerous hospitals to help with participant recruitment [35]. Due to the nature of the disease, there is the issue of accessing sufficient numbers of healthcare users as well as appropriately experienced healthcare professionals to create meaningful outcome measure prioritisation. Perhaps recruiting through the National Health Service (NHS) may prove more useful to identify healthcare users, but could also reduce international stakeholder collaboration. Additionally, an e-Delphi may not be the best methodology to recruit sufficient numbers of healthcare professionals from within such a restricted pool of a rare disease. This could ultimately necessitate prolonged intervals between Delphi rounds to optimise participation and retention. Participants were not remunerated for their involvement in the e-Delphi or consensus meeting, which may have been another contributing factor to the lack of engagement, significantly for healthcare users. Both healthcare users and providers within rare diseases have very limited spare time to be able to set aside additional tasks. Providing such incentives could promote greater engagement in such exercises.

**Fig. 3:**
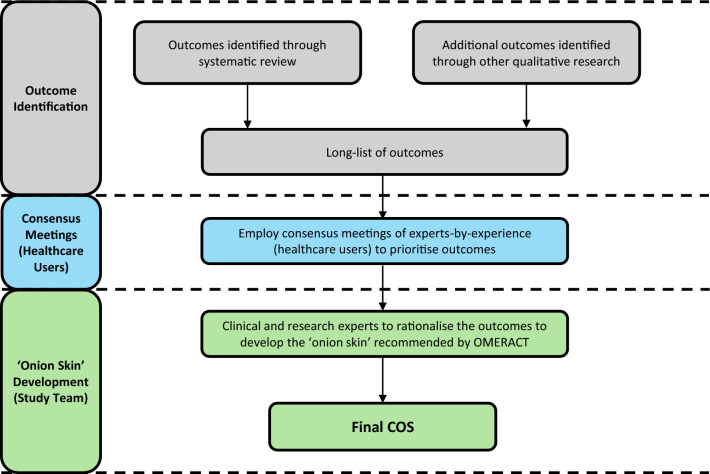
Recommended methodology for determining a core outcome set for a rare disease

Typically, a Delphi methodology involves two or more rounds of key stakeholder voting followed by a face-to-face meeting with representatives of the key stakeholder groups to determine the final COS [20]. Our work differed in that one stakeholder group (healthcare providers) opinion was gathered during the Delphi process followed by a consensus meeting with another stakeholder group (healthcare users) to gain their opinions and to further ratify the list of outcomes. Outcomes were then rationalised by clinical and research experts. Although the work conducted does not stray massively from COMET principles, based on our work and experience, a modified methodology is recommended when producing a COS for rare diseases (Fig. 3). Firstly, develop a long-list of candidate outcomes though a preceding systematic review of the literature and/or qualitative semi-structured interviews. Secondly, a consensus meeting(s) would be held for healthcare users (experts-by-experience) to help prioritise the identified outcomes. Finally, clinical and research experts then rationalise the prioritised outcomes and categorise as recommended by the OMERACT group, maintaining healthcare users’ priorities of outcomes. Utilising their understanding of existing outcomes measurement instruments and the impact of the disease to distribute outcomes within the onion layers. This methodology ensures that the opinions of both professional experts and experts-by-experience contribute to the COS. Consensus meetings should benefit from the involvement of a rare disease charity due to the crucial role that these organisations play in communication between healthcare users and professionals.

## Conclusion

The COHERE study has developed the first COS to be used in clinical trials relating to MPS II. Identified mandatory outcomes for patients aged 0 to 25 years include: quality of life, survival, and airway obstruction (Fig. 2). The standardisation of outcome measurement in rare diseases is crucial to allow the amalgamation of data between studies and reduce research waste. Ultimately, this will facilitate the reporting of therapeutic interventions and hence the translation of therapeutic research into evidence-based treatments.

Healthcare user and professionals’ participation has been a major barrier to COS generation in MPS II, therefore, collaboration with charities and social enterprises representing healthcare users is crucial to facilitate adequate recruitment. The COHERE group recommends developing a long-list of candidate outcomes, followed by consensus meeting(s) with experts-by-experience to prioritise outcomes, with final rationalisation of outcomes by clinical and research experts to categorise within the recommendations of the OMERACT group.

## Data Availability

The datasets used and/or analysed during the current study are available from the corresponding author on reasonable request.

## Appendix 1

The long list of 46 candidate outcomes identified by systematic review and qualitative interviews, and their plain language definition.

**Table:**

Dodd taxonomy core area	Outcome	Plain language definition
Mortality/Survival	Survival	Continuing to be alive
Physiological/clinical	Blood gas	Levels of gases in the bloodstream (e.g., oxygen and carbon dioxide)
	Acute Otitis Media (AOM)	Infection in the middle ear space behind the eardrum
	Tinnitus	Abnormal noises (e.g., ringing or buzzing) in the ear
	Tympanic membrane perforation	Hole in the eardrum
	Vertigo	Sensation of imbalance, usually spinning
	Chronic Otitis Media (COM)	Problems in the middle ear space behind the eardrum (e.g., infection) that last more than 3 months
	Middle ear function	The ability to change the conditions (pressure) behind the ear drum (in the enclosed air-filled middle ear space) to match the surroundings
	Hearing	Ability to hear noises
	Purulent ear discharge	Pus from the ear due to infection (also called otorrhoea)
	Temporal bone status	Changes on a CT scan of the ear that might be linked to ear problems
	Pulmonary function	How the lungs are working
	Sleep apnoea	Breath holding during sleep
	Chronic nasal discharge	Discharge from the nose lasting weeks or months
	Airway obstruction	Blockage or narrowing of the air passages that may occur anywhere from ‘lips to lungs’
	Dyspnoea	Shortness of breath
	Chronic cough	Cough lasting more than 8 weeks
	Bronchitis	Infection or inflammation of the large airways of the lungs
	Pneumonia	Infection of the lungs
	Enlarged tonsils	The lumps of tissue at the back of the throat called ‘tonsils’ being larger than normal
	Enlarged adenoids	The lump of tissue at the back of the nose called ‘adenoids’ being larger than normal
	Swallowing difficulties	Problems with swallowing liquids or solids
	Nasal congestion/ obstruction	Blocked nose
	Rhinorrhoea	Discharge from the nose lasting weeks or months
	Nasal polyps	Fluid filled sacs that grow in the nose in response to inflammation, that can cause nasal congestion, runny nose and poor sense of smell
	Rhinosinusitis	Inflammation or infection in the nose and sinuses, that can lead to facial pain, blocked nose and nasal discharge
Life impact	Exercise tolerance	How much exercise you are able to do before stopping
	Language development	The process of progressing from ‘babbling’ and using single words through to speaking in detailed sentences
	Communication skills	How well you can make other people understand you
	Intelligence	How clever you are
	Psycho-social development	How well you learnt to make friends and speak to new people
	Quality of life (QoL)	The standard of health, comfort, and happiness experienced by an individual or group
	Ability to attend school and college, or to seek employment	Being able to attend a place of education or work, and/or participate in educational activities or work
	Education	How well they are doing at school or college
	Emotional impact if the disease on the patient	Effect of disease on my feelings
	Emotional impact of the disease on parents/carers	Effect of disease on feelings of parents and carers
	Independence	Impact of ear, nose and throat and lung/breathing problems on independence
Resource use	Need for surgical intervention to ears	Needing to have an operation
	Need for non-invasive ventilatory support	Needing to wear a mask to support breathing by increasing the pressure or flow of gases to and from the lungs
	Non-surgical intervention to ears	A therapy provided to improve how the ears work
	Tonsillectomy	Surgical removal of the tonsils from the back of the throat
	Adenoidectomy	Surgical removal of the adenoids from the back of the nose
	Tracheotomy	Surgical airway that involves breathing through a tube inserted into the windpipe through the front of the neck
	Need for oxygen therapy	Needing to be given additional oxygen as part of medical treatment
	Prevalence of pulmonary-related hospitalizations	The need to be admitted to hospital to manage lung problems
Adverse events/effects	Ability to secure the airway by endotracheal intubation	How easy it is for a doctor to pass a tube into the windpipe via the mouth, to support breathing during a general anaesthetic or period on intensive care
	Behaviour	How a person acts (e.g. cooperative or aggressive), especially towards other people
	Sleep quality	How peaceful is the person’s sleep
	Voice quality	How the voice sounds
	Complication of surgical treatment	Negative health problem that develops as a result of a surgical operation

## Appendix 2

Content of the e-Delphi survey.

*This text will appear on home Page of the Delphi survey, if participants agree with these statements they will progress with registration.*

- I confirm I have read and understood the participant information sheet dated 03 Feb 2020 (version 1.1) for the COHERE study.
- I understand that my participation is voluntary and that I am free to withdraw at any time, without giving any reason, without my medical care or legal rights being affected.
- I understand that there are 2 rounds of the survey and that I agree to be contacted to take part in round 2.
- I agree to be contacted about the results of the study.
- I agree to be contacted to take part in future stages of the COHERE study.
- I understand that relevant sections of data collected during the study may be looked at by responsible individuals from the University of Manchester, from regulatory authorities or from Manchester University Foundation Trust, where it is relevant to my taking part in the research. I give permission for these individuals to have access to this data.
  If you agree with these statements please register to take part in the study.

*Dependent upon the stakeholder group, the participant was asked to confirm and enter information from the below*.

**Table:**

Stakeholder	Recorded participant demographic information
Healthcare users	Gender (male/female/other/prefer not to say)
	Age
	Confirmation of MPS II diagnosis (Yes/No)
	Country of residency
Parents and/or carers	Confirmation of being a parent of a child with MPS II (Yes/No)
	Number of children with MPS II
	Ages of children with MPS II
	Country of residency
Healthcare professionals	Profession (ENT surgeon, respiratory physician, clinical geneticist, metabolic physician, specialist nurse, physiotherapist, general paediatrician, audiologist, other (please state))
	Job title
	Years since qualified
	I confirm I have looked after at least 2 patients with MPS II in the last 12 months (Yes/No)
	Country of residency
